# Paper Towels for Surgical Purposes

**Published:** 1885-10

**Authors:** 


### Paper Towels for Surgical Purposes.

Dr. Roberts, of Philadelphia, has been using with much satisfaction Japanese paper handkerchiefs for drying wounds. Sponges are so seldom and with such difficulty perfectly cleansed after being once used, that they are never employed in the clinic. Ordinary cotton or linen towels are much preferable to sponges, but if dirty are liable to introduce septic material into wounds. The paper towels answer the same purposes as cotton ones, and are so cheap that they can be thrown away after being used. They cost from $6 to $7.50 a thousand. The cost of washing a large number of ordinary towels is thus avoided. The paper towels are scarcely suitable for drying hands, after washing, unless several towels are used at once, because the large amount of moisture on the hands soon saturates a single towel. For removing blood from wounds, a paper towel is crumpled up into a sort of ball and then used as a sponge. Such balls absorb blood rapidly. The crude ornamental pictures in color on the towels are of no advantage, nor are they, as far as known, any objection.—*Exchange.*

